# Cross-cultural adaptation of the Neck Disability Index and Copenhagen Neck Functional Disability Scale for patients with neck pain due to degenerative and discopathic disorders. Psychometric properties of the Polish versions

**DOI:** 10.1186/1471-2474-12-84

**Published:** 2011-04-29

**Authors:** Ewa Misterska, Roman Jankowski, Maciej Glowacki

**Affiliations:** 1 Department of Pediatric Orthopaedics and Traumatology, Poznan University of Medical Sciences, ul. 26 Czerwca 1956, Poznan, Poland; 2 Department of Neurosurgery and Neurotraumatology, Poznan University of Medical Sciences, ul. Przybyszewskiego 49, Poznan, Poland

## Abstract

**Background:**

Even though there are several region-specific functional outcome questionnaires measuring neck disorders that have been developed in English-speaking countries, no Polish version has ever been validated. The purpose of our study was to translate, culturally adapt and validate the Neck Disability Index (NDI) and Copenhagen Neck Functional Disability Scale (CDS) for Polish-speaking patients with neck pain.

**Methods:**

The translation was carried out according to the International Quality of Life Association (IQOLA) Project. Sixty patients were treated due to degenerative and discopathic disorders in the cervical spine filled out the NDI-PL and the CDS-PL. The pain level was evaluated using the Visual Analog Scale. The mean age of the assessed group was 47.1 years (SD 8.9). We used Cronbach's alpha to assess internal consistency. We assessed the test-retest reliability using the Intraclass Correlation Coefficients (ICCs). The Spearman's rank correlation coefficient (rS) was used to determine dependency between quantitative characteristics. The Mann-Whitney test was applied to determine dependency between quantitative and qualitative characteristics.

**Results:**

The Cronbach's alpha values were excellent for the NDI-PL in the test and in the retest (0.84, 0.85, respectively), and for the CDS-PL (0.90 in the test and in the retest). Intraclass Correlation Coefficients were excellent for the CDS-PL and NDI-PL and equalled 0.93 (95% CI from 0.89 to 0.95) and 0.87 (95% CI from 0.80 to 0.92), respectively The concurrent validity was good in the test and in the retest (rs = 0.42 p < 0.001; rs = 0.40 p = 0.002, respectively) for NDI-PL and for CDS-PL (rs = 0.42 p < 0.001; rs = 0.40 p = 0.001, respectively). The adapted questionnaires showed a strong inter-correlation both in the test (0.87 p < 0.001) and in the retest (0.79 p < 0.001).

**Conclusions:**

The present versions of the NDI-PL and CDS-PL, the first to be published in Polish, have proven to be reliable and valid for patients with degenerative changes in the cervical spine. The NDI-PL and CDS-PL have excellent internal consistency and test-retest reliability, and good concurrent validity. The adapted questionnaires showed a strong inter-correlation both in the test and in the retest. No ceiling or floor effects were detected in the NDI-PL and CDS-PL. The NDI-PL and CDS-PL are comparable with other versions and can be recommended and used in international comparative studies.

## Background

Annually about 30% of the population experience neck pain (NP), 14% of whom report complaints lasting longer than 6 months [1,2]. Many authors' experiences indicate that subjective assessment of pain intensity and how it influences the extent to which everyday activities are executed is becoming increasingly significant and an important component of clinical practice, however it is vital that, the tools used in the evaluation of the level to which everyday activities are carried out are valid and reliable [1].

There are several questionnaires available measuring NP that have been developed and published in English-speaking countries: Neck Disability Index (NDI) [3,4], Neck Pain and Disability Scale (NPDS) [5], the Northwick Park Neck Pain Questionnaire (NPNPQ) [6], the Copenhagen Neck Functional Disability Scale (CDS) [7]. These are classed as region-specific functional outcome questionnaires, which concentrate on specific parts of the body, therefore providing more detailed data on its function within a defined disease entity with greater responsiveness compared to a questionnaire of a general nature such as the Short Form-36 [SF-36] [8]. SF-36 is a multi-purpose, short-form health survey with 36 questions and yields an 8-scale profile of functional health and well-being scores as well as psychometrically-based physical and mental health summary measures [8].

A number of authors [6,9-11] highlight the need to adapt recognized and widely applied assessment tools in research rather than developing a new scale leading to the multiplication of outcome measures lacking the comparison of populations [1].

We decided to evaluate the Polish versions of the Copenhagen Neck Functional Disability Scale (CDS-PL) and the Neck Disability Index (NDI-PL). The NDI is the scale most commonly applied, extensively tested and translated. The English version of the NDI has shown moderate differences in reliability and validity with different patient populations [12]. The responsiveness of the NDI is unknown, however concurrent validity when compared with the Visual Analog Scale has been reported [4].

The NDI has been shown to be a valid and reliable instrument to measure disability related to neck pain in studies conducted for French, Brazilian-Portuguese, Iranian, Greek, Finnish, Spanish, Turkish, Korean, Dutch, Chinese, Swedish-speaking patients [1,13-21]. The CDS focuses on quality of life and the scores for each item as rated by the patient can be easily transferred and interpreted in terms of their clinical relevance [7,22]. Despite its many advantages, to our knowledge, the CDS has only been successfully translated and validated into French [22].

The objective of this prospective study was to translate and culturally adapt the NDI and CDS into Polish and to validate their use among Polish-speaking patients with NP. To our knowledge, no questionnaire assessing disability in everyday activities in Polish-speaking patients with neck pain has ever been evaluated and tested for its psychometric properties. Our hypothesis was that if we adapt the NDI and CDS to the Polish cultural conditions and test psychometric properties of the NDI-PL and CDS-PL, such as internal consistency, test-retest reliability, concurrent validity, ceiling or floor effects and analyses of the item-total correlation, then we will achieve assessment tools that are equivalent to the original English- language questionnaires. As a result, we aimed to achieve tools that would help us properly assess pain intensity and the related limitations of cervical spine function during the execution of everyday activities in Polish conditions.

## Methods

The NDI questionnaire was designed by Vernon and Mior in 1992 to assess pain intensity and the related limitations of cervical spine function during the execution of everyday activities [4]. The NDI is based on the Oswestry Disability Index and is composed of 10 questions: pain intensity, personal care, lifting, reading, headaches, concentration, work, driving, sleeping, and recreation [4]. Each item is scored from 0 (no disability) to 5 (total disability). The maximum possible score is 50. However, the sum of the scores obtained is often doubled to give a percentage score out of 100. The interpretation is as follows: 0-20 normal, 21-40 mild disability, 41-60 moderate, 61-80 severe and 80 or over (complete or exaggerated disability). Since the questionnaire is straightforward, the average patient needs approximately 5 minutes to complete it [4,23].

The CDS consists of 15 items that evaluate the impact of neck pain. Three items evaluate pain severity directly, including the patient's perception of the future impact of neck pain, eight items evaluate disability during everyday activities and four items focus on social interaction and recreation [7,22]. There are three possible answers to select from each item; "yes" (2 points), "occasionally" (1 point), and "no" (0 points). For items 1 - 5 however the scoring is reversed and here "yes" carries a score of 0, "occasionally" 1 and "no" 2. The highest score attainable is 30, indicating worst possible impact, the lowest is 0 where no impact of neck pain can be identified. 90 seconds is invariably sufficient in order to complete the questionnaire [7].

### Translation procedure

The translation was carried out in accordance with the recommendations proposed by Beaton et al. [9]. In the first stage two independent translators, of whom one had a medical background, translated the original versions of the NDI and CDS into Polish. In the second stage, a team comprised of the project authors and both translators compared and synthesised the translations. In the third phase, two bilingual translators performed the back-translation where the Polish versions of the questionnaires were translated into the original language. In the fourth stage, the expert committee reviewed all translations and created a prefinal version of the questionnaires. In order to evaluate the psychometric properties of the questionnaire, 60 patients who fulfilled the inclusion criteria for the study completed the NDI-PL, CDS-PL and 100-mm Visual Analogue Scale twice. (See additional file 1: Copenhagen Neck Functional Disability Scale_Polish version and additional file 2: Neck Disability Index_Polish version).

### Evaluation of the psychometric properties of the NDI-PL and CDS-PL

1. We analyzed means, minimal and maximal values, standard deviations and 95% confidence intervals for the general results, for the CDS-PL dimensions and for the NDI-PL questions.

2. We analyzed floor and ceiling effects (% of patients with the minimal score and % of patients with the maximum score). Ceiling and floor effects are considered to be present if more than 15% of respondents achieved the lowest or highest possible total score [24].

3. We used Cronbach's alpha to assess internal consistency. Additionally, we performed analyses of the item-total correlation for the NDI-PL and CDS-PL.

4. We analyzed the correlations between the NDI-PL and CDS-PL.

5. We performed an assessment of the test-retest reliability using the Intraclass Correlation Coefficients (ICCs), type 2.1. The NDI-PL and CDS-PL were completed twice at a 24-hour interval.

6. For construct-related validity, the concurrent validity method was used. To examine the concurrent validity, the relation between the NDI-PL and CDS-PL and 100-mm Visual Analogue Scale was examined by the Spearman's rank correlation coefficient.

Statistical analysis was carried out using the Statistica program. In the quality field we supplied the number of units for specific categories of a given characteristic and their relative percentage values. The Spearman's rank correlation coefficient (rS) was used to determine dependency between quantitative characteristics. The Mann-Whitney test was applied to determine dependency between quantitative and qualitative characteristics.

The borderline value of statistical significance was set at p = 0.05. Test results with a greater value than this were deemed to be statistically irrelevant. Cronbach's alpha values were accepted as follows: ≥0.80 as excellent, 0.70-0.79 as adequate and < 0.70 as poor [25].

Concurrent validity coefficients were accepted as follows: rS = 0.81-1.0 as excellent, 0.61-0.80 very good, 0.41-0.60 good, 0.21-0.40 fair, and 0-0.20 poor [23,25]. Values of Intraclass Correlation Coefficient (ICC) above 0.80 were considered as evidence of excellent reliability [12].

### Participants

Patients eligible for the study were consecutively recruited from June 2009 to September 2010. Eligibility criteria were the following: written consent of the patient, neck pain lasting more than 3 months; ability to read and speak Polish fluently; age 18-60 years. MRI of cervical segments of the spine was carried out in all cases. The analysis did not include pregnant woman and patients suffering from spinal tumors, vertebral traumatic fractures, neurological and psychiatric disorders causing difficulty in speech communications. All physical examinations were performed by the same physician, a neurosurgeon. All patients were operated on due to discopathy and vertebral degenerative changes in the cervical spine. Surgery was carried out via anterior vertebral approach and consisted of the decompression of the spinal cord and subsequent arthrodesis. Clinical state was determined before surgery in our study.

All examined persons were guaranteed anonymity and written consent was required. Demographic variables and the previous history of disease were taken from all of the patients. Disability was evaluated with the NDI-PL and the CDS-PL. The pain level was evaluated using 100 mm Visual Analogue Scale. Table 1 summarizes the demographic and clinical characteristics of the patients.

**Table 1:** Demographical and clinical characteristics of study participants (n = 60)

Variable	All study participants *	Range**
Gender		

Male	26 (43.3%)	---

Female	34 (56.7%)	---

Age (yr)	47.1 (8.9)	28-60

Weight (kg)	73.2 (15.7)	40-122

Height (cm)	168.4 (8.8)	155-192

Neck pain duration (months)	41.8 (60.0)	3-360

Neck pain intensity (VAS) (mm)	47.0 (23.0)	1-90

Neck pain intensity after 2 days (VAS) (mm)	48.3 (22.7)	1-90

Number of discopathy levels		

1 level	20 (33.3%)	---

2 or more levels	40 (66.7%)	---

Changes of signal intensity in spinal cord in MRI	13 (21.7%)	---

Sagittal dimension of vertebral canal on the discopathy level		

> 9 mm	28 (46.7%)	---

≤ 9 mm	32 (53.3%)	---

Symptoms		

Local cervical (neck) pain	9 (15.0%)	---

Cervicobrachialgia	26 (43.3%)	---

Cervicobrachialgia and melopathy	25 (41.7%)	---

*Continuous data are mean (SD); categorical data are N (%)

**Range (min-max) for continuous data

Local cervical spinal pain was found in 9 patients, cervicobrachialgia in 26 patients and cervicobrachialgia and myelopathy in 25 participants. Intensity of pain, determined in the 100-mm Visual Analogue Scale, was 47.0 mm (SD 23.0), range 1-90 during the first examination and 48.3 mm (SD 22.7), range 1-90 in the second. Additional information on the study group can be seen in Table 1.

### Ethical issues

The study design was approved by the Bioethics Commission (approval number 744/09) and was carried out following universal ethical principles.

## Results

### Distribution of the results

Mean scores and standard deviations, the minimum, maximum, and 95% confidence intervals were calculated for both administrations of the NDI-PL and CDS-PL. The mean value of the NDI-PL general result equalled 21.6 (SD 7.9) in the test and 20.6 (SD 8.0) in the retest, which is interpreted as normal and mild disability, respectively. The mean value of CDS-PL general result was 17.7 out of 30 possible points (SD 8.3) in the test and in the retest (Table 2).

**Table 2:** Distribution of minimal and maximal scores, mean scores, 95% confidence interval in NDI-PL and CNFDS-PL

			Test						Retest			
	**Min**	**Max**	**Mean** **value**	**95%** **Confidence** **interval**		**SD**	**Min**	**Max**	**Mean** **value**	**95%** **Confidence** **interval**		**SD**

				**from**	**to**					**from**	**to**	

NDI-PL												

Total score	3	36	21.6	19.6	23.6	7.9	3	42	20.6	18.5	22.6	8.0

Pain intensity	0	5	2.1	1.8	2.3	1.0	0	5	2.0	1.7	2.2	1.0

Personal care	0	4	1.3	1.0	1.5	0.9	0	4	1.2	1.0	1.4	0.9

Lifting	0	5	2.6	2.2	3.0	1.6	0	5	2.6	2.2	2.9	1.4

Reading	0	4	2.4	2.2	2.7	1.0	0	5	2.4	2.1	2.7	1.2

Headaches	0	5	2.4	2.1	2.8	1.4	0	5	2.1	1.8	2.5	1.4

Concentration	0	4	1.5	1.2	1.7	1.0	0	5	1.4	1.1	1.7	1.0

Work	0	5	2.6	2.3	2.9	1.2	0	5	2.4	2.1	2.7	1.2

Driving	0	5	2.8	2.3	3.2	1.4	0	5	2.7	2.3	3.1	1.3

Sleeping	0	5	2.2	1.9	2.5	1.2	0	5	2.1	1.7	2.5	1.4

Recreation	0	5	2.5	2.2	2.9	1.3	0	5	2.4	2.0	2.7	1.3

CDS-PL												

Total score	0	30	17.7	15.5	19.8	8.3	0	30	17.7	15.6	19.9	8.3

Pain severity	0	6	3.9	3.4	4.4	1.9	0	6	3.4	3.4	4.5	2.1

Disability	0	16	9.0	7.9	10.1	4.4	0	16	7.6	7.6	9.9	4.3

Social interaction	0	8	4.8	4.1	5.5	2.7	0	8	4.3	4.3	5.7	2.7

NDI-PL-Polish language version of the Neck Disability Index; the score range: 0-100%

CDS-PL- Polish language version of the Copenhagen Neck Functional Disability Scale

### Floor and ceiling effect

We have analyzed floor and ceiling effects for the general results of the CDS-PL and NDI-PL. In the case of CDS-PL, in both the test and retest, 3.3% of patients received the minimum score (2 participants), and the 1.7% of patients received the maximum score (1 participant). Patients with the minimum and maximum score were not identified in the test nor in the retest of the general result of the NDI-PL. Both in the CDS-PL and NDI-PL floor or ceiling effects were not detected as less than 15% achieved the minimum or maximum possible scores.

### Internal consistency

Table 3 presents the Cronbach's alpha values, concurrent validity in the test and in the retest and test-retest reliability measured by the Pearson correlation coefficient. The Cronbach's alpha values are excellent for the NDI-PL in the test (0.84) and in the retest (0.85). Cronbach's alpha values for the CDS-PL are excellent, and equalled 0.90 in the test and in the retest (Table 3). Moreover, the analyses of item-total correlation confirmed that both scales are internally consistent (Table 4 and Table 5).

**Table 3:** Cronbach's alpha, criterion validity and test-retest reliability values for the NDI-PL and CDS-PL

		Test			Retest		Test-retest reliability
	**Cronbach's alpha**	**Concurrent validity**	**Correlation between NDI-PL and CNFDS-PL**	**Cronbach's alpha**	**Concurrent validity**	**Correlation between NDI-PL and CNFDS-PL**	

NDI-PL	0.84	rs = 0.42 p < 0.001	0.87	0.85	rs = 0.40 p = 0.002	0.79	ICC = 0.87 95% CI from 0.80 to 0.92

CDS-PL	0.90	rs = 0.42 p < 0.001	p < 0.001	0.90	rs = 0.40 p = 0.001	p < 0.001	ICC = 0.93 95% CI from 0.89 to 0.95

NDI-PL- Polish language version of the Neck Disability Index

CDS-PL- Polish language version of the Copenhagen Neck Functional Disability Scale

**Table 4:** Item-total correlation analyses for the NDI-PL

Questions of NDI	Test		Retest	
	**rs**	**p value**	**rs**	**p value**

Pain intensity	0.60	<0.001	0.59	<0.001

Personal care	0.60	<0.001	0.71	<0.001

Lifting	0.73	<0.001	0.69	<0.001

Reading	0.70	<0.001	0.81	<0.001

Headaches	0.43	<0.001	0.56	<0.001

Concentration	0.76	<0.001	0.73	<0.001

Work	0.68	<0.001	0.72	<0.001

Driving	0.63	<0.001	0.65	<0.001

Sleeping	0.68	<0.001	0.59	<0.001

Recreation	0.77	<0.001	0.62	<0.001

NDI-PL- Polish language version of the Neck Disability Index

**Table 5:** Item-total correlation analyses for the CNFDS-PL

Questions of CDS	Test		Retest	
	**rs**	**p value**	**rs**	**p value**

1. Can you sleep at night without neck pain interfering?	0.61	<0.001	0.72	<0.001

2. Can you manage daily activities without neck pain reducing activity levels?	0.69	<0.001	0.78	<0.001

3. Can you manage daily activities without help from others?	0.61	<0.001	0.52	<0.001

4. Can you manage putting on your clothes in the morning without taking more time than usual?	0.65	<0.001	0.65	<0.001

5. Can you bend over the washing basin in order to brush your teeth without getting neck pain?	0.64	<0.001	0.73	<0.001

6. Do you spend more time than usual at home because of neck pain?	0.71	<0.001	0.69	<0.001

7. Are you prevented from lifting objects weighing from 2-4 kilograms due to neck pain?	0.64	<0.001	0.56	<0.001

8. Have you reduced your reading activity due to neck pain?	0.56	<0.001	0.59	<0.001

9. Have you been bothered by headaches during the time that you have had neck pain?	0.46	<0.001	0.38	0.003

10. Do you feel your ability to concentrate is reduced due to neck pain?	0.58	<0.001	0.70	<0.001

11. Are you prevented from participating in your usual leisure time activities due to neck pain?	0.50	<0.001	0.52	<0.001

12. Do you remain in bed longer than usual due to neck pain?	0.68	<0.001	0.59	<0.001

13. Do you feel that neck pain has influenced your emotional relationship with your nearest family?	0.64	<0.001	0.70	<0.001

14. Have you had to give up social contact with other people during the past two weeks due to neck pain?	0.68	<0.001	0.65	<0.001

15. Do you feel that neck pain will influence your future?	0.62	<0.001	0.65	<0.001

CDS-PL- Polish language version of the Copenhagen Neck Functional Disability Scale

### Concurrent validity

The concurrent validity of NDI-PL measured by the Spearman's rank correlation coefficient was good in the first test and in the retest (rS = 0.42, p < 0.001; rS = 0.40, p = 0.002, respectively). The concurrent validity of the CDS-PL was good in the test and in the retest (rS = 0.42, p < 0.001; rS = 0.40, p = 0.001, respectively) (Table 3).

### Test-retest reliability

Intraclass Correlation Coefficients were excellent for the CDS-PL and NDI-PL and equalled 0.93 (95% CI from 0.89 to 0.95) and 0.87 (95% CI from 0.80 to 0.92), respectively (Table 3).

### The inter-relationships

Both in the test (0.87 p < 0.001) and in the retest (0.79 p < 0.001), the adapted questionnaires exhibited a strong inter-correlation (Table 3).

Additional information on the correlation between the selected clinical patients characteristics and general results of NDI-PL and CDS-PL can be seen in Table 6.

**Table 6:** Correlation between the selected clinical patients characteristics and general results of NDI-PL and CDS-PL

	Number of discopathy levels	Changes in signal intensity in spinal cord in MRI	Sagittal dimension of vertebral canal on the discopathy level	Neck pain duration
		**Test**		

CDS-PL	p = 0.660	p = 0.029*	p = 0.132	r_S _= 0.17 p = 0.172

NDI-PL	p = 0.632	p = 0.044*	p = 0.023*	r_S _= 0.19 p = 0.137

		**Retest**		

CDS-PL	p = 0.759	p = 0.059	p = 0.145	r_S _= 0.11 p = 0.378

NDI-PL	p = 0.814	p = 0.193	p = 0.350	r_S _= 0.19 p = 0.127

NDI-PL- Polish language version of the Neck Disability Index

CDS-PL- Polish language version of the Copenhagen Neck Functional Disability Scale

### The correlation between selected patient clinical characteristics and the results of the NDI-PL and CDS-PL

We have also assessed the correlation between selected patient clinical characteristics and the results of the adapted assessment tools. The only statistically significant correlations were identified between CDS-PL and changes in signal intensity in spinal cord in MRI (p = 0.29) and between NDI-PL and changes in signal intensity in spinal cord in MRI (p = 0.44) and the sagittal dimension of the vertebral canal on the discopathy level (p = 0.23), in the first completion of the questionnaires (Table 6).

The present results confirmed our hypothesis. We have proved that the NDI-PL and CDS-PL have excellent internal consistency and test-retest reliability, good concurrent validity and showed a strong inter-correlation both in the test and in the retest. Moreover, no ceiling or floor effects were detected in the NDI-PL and CDS-PL.

## Discussion

To our knowledge, this is the first study of a Polish version of a questionnaire assessing disability in everyday activities in patients with neck pain. Even though there are several region-specific functional outcome questionnaires measuring neck disorders available, no Polish version has ever been validated. Our study indicated that NDI-PL and CDS-PL are valid and reliable methods for measuring disability in Polish patients with neck pain.

There have been numerous studies on the reliability and validity of the Neck Disability Index for patients with neck pain [3,4,23]. Vernon and Mior obtained a high degree of test-retest reliability in patients with post-traumatic neck pain, using the Pearson correlation coefficient [4].

The internal consistency of each questionnaire was assessed using Cronbach's alpha. We also assessed the item-total correlations. Our study indicated that NDI-PL and CDS-PL are internally consistent. In comparison to the French version of CDS [22], where all items had good or fair correlations with the total score, almost all items of CDS-PL had very good correlations with the general result. The Cronbach's alpha coefficient of the total score of CDS-PL was excellent and higher, compared to the French version of CDS. However, the Cronbach's alpha in our study was exactly the same as in the original version of the CDS (0.90) [7].

Excellent internal consistency of the NDI-PL (0.90) is comparable with the results from other studies - Spanish, Finnish, Iranian or Brazilian versions of NDI [13,14,16,17]. It is higher than the value obtained by Vernon and Mior (0.8) in the original NDI [4]. Pearson correlation coefficients between individual NDI-PL item scores and the total ranged from 0.43 to 0.77 (test) and from 0.56 to 0.81 (retest) and, as in the original version of the NDI [3,4] no item dominated with an especially high correlation and no item appeared to be redundant.

We assessed the test-retest reliability of NDI-PL and CNFDS-PL using the Intraclass Correlation Coefficients (ICCs). We decided to choose a retest interval of 2 days, similarly to the authors of the Spanish or Iranian version of NDI, in order to avoid variations in the clinical status of the patients and to avoid the patients remembering their previous answers. As suggested by Holt et al. [26], a long interval period may be inappropriate for a test-retest study of health measures because too many changes in the patient's health status can occur. Opinions regarding the appropriate interval have varied from 1 hour to 1 year, but a retest interval of 2 to 14 days is generally accepted [27]. The very good test-retest reliability of NDI-PL is comparable with the results of the Iranian (0.90) and Greek (0.93) versions of the NDI [16,19]. Our findings are also similar to the original version of NDI (0.90) [3]. Furthermore, the NDI-PL test-retest reliability values were found to be slightly higher than those achieved during the initial trials for the original version when study participants completed the questionnaires with an interval of 2 days between the first and second test as occurred in our study (0.80) [4].

For the NDI-PL and CDS-PL the expected good concurrent validity was observed. As in the Spanish version of the NDI, a good correlation with the VAS score is evident, which may signify, as highlighted by Andrade et al [13], that the NDI is designed to assess not pain levels as such but rather disability due to pain experienced. The results of our study showed that the correlation between NDI-PL and CDS-PL is very high, showing a clear association between these two measures, which seem to measure similar constructs.

What is more, our examinations proved that the degree of spinal cord compression and spinal cord ischemic changes, expressed as changes of MRI signal intensity, correlate with the disability scales NDI-PL and CDS-PL. Intervertebral cervical disc herniation and degenerative changes are associated with narrowing of the sagital diameter of the spinal canal. Progressive compression may lead to spinal cord ischemia, leading to histopathological changes of the spinal cord. Nonetheless, these changes may or may not be symptomatic [28,29].

### Limitations

Our population was limited to patients with degenerative and discopathic disorders in the cervical spine, which may limit the generalizability of the findings to other populations. Fifteen (25%) of the patients participating in our study omitted the section concerned with driving (section 8). This is consistent with both the Dutch and Turkish versions of the NDI, where 21% and 23.87% of participants did not answer this section [1,5]. It was not necessary to modify this section as the number of patients who omitted it was low. The study evaluating the Greek version of the NDI recorded that 44.6% of patients decided not to answer the questions on 'driving' [19].

### Future research

Despite the fact that we confirmed that both adapted assessment tools have excellent internal consistency, test-retest reliability, and good concurrent validity, further investigation is required to provide additional data for the evaluation of the psychometric properties of the NDI-PL and CDS-PL. In future studies responsiveness, which is a useful property required for determining if the measures are sensitive to detect changes over time, should be tested. Likewise, the item-level analyses of the NDI-PL and CDS-PL would be helpful in future research to provide a more detailed analysis of the functioning of the items for the population.

## Conclusion

Our study presents an analysis of the psychometric properties of two region-specific functional disability scales for patients with degenerative changes in the cervical spine. As far as we know, this article describes the first attempt at translation and validation of questionnaires appropriate for Polish-speaking patients with neck pain. We have indicated, that the present versions of the NDI-PL and CDS-PL, the first to be published in Polish, have proven to be reliable and valid.

The NDI-PL and CDS-PL have excellent internal consistency and test-retest reliability, and good concurrent validity. The adapted questionnaires showed a strong inter-correlation both in the test and in the retest. No ceiling or floor effects were detected in the NDI-PL and CDS-PL. The NDI-PL and CDS-PL are comparable with other versions and can be recommended and used in international comparative studies.

## Competing interests

The authors declare that they have no competing interests.

## Authors' contributions

EM participated in the statistical analysis, data interpretation and manuscript preparation, RJ participated in the collection of data, data interpretation and manuscript preparation, MG participated in the design of the study, data interpretation, manuscript preparation

All authors read and approved the final manuscript.

## Pre-publication history

The pre-publication history for this paper can be accessed here:

http://www.biomedcentral.com/1471-2474/12/84/prepub

## Supplementary Material

Additional file 1 **Copenhagen Neck Functional Disability Scale_Polish version**. Polish language version of adapted Copenhagen Neck Functional Disability Scale Click here for file

Additional file 2 **Neck Disability Index_Polish version**. Polish language version of adapted Neck Disability Index Click here for file
