# Supplementary material for: Cross-cultural adaptation of the Neck Disability Index and Copenhagen Neck Functional Disability Scale for patients with neck pain due to degenerative and discopathic disorders. Psychometric properties of the Polish versions
Source: BMC Musculoskelet Disord. 2011 Apr 29;12:84. doi: 10.1186/1471-2474-12-84 (PMC3108936; doi:10.1186/1471-2474-12-84)
Supplement: Additional file 1 — Copenhagen Neck Functional Disability Scale_Polish version. Polish language version of adapted Copenhagen Neck Functional Disability Scale [file 1471-2474-12-84-S1.PDF]

KOPENHASKA SKALA NIESPRAWNOŚCI CZYNNOŚCIOWEJ CZĘŚCI SZYJNEJ KRĘGOSŁUPA

THE COPENHAGEN NECK FUNCTIONAL DISABILITY SCALE-POLISH VERSION

Imię i nazwisko pacjenta: ____________________________________Data: ___________

Proszę uważnie przeczytać:

Niniejszy kwestionariusz został opracowany, aby pomóc nam zrozumieć, jak bardzo dolegliwości bólowe szyi wpływają na Państwa zdolność wykonywania codziennych czynności. Odpowiadając na pytania, proszę zaznaczyć TYLKO jedną odpowiedź dla każdego pytania.

**OBJAW**	TAK	CZASAMI	NIE
1. Czy możesz spać w nocy bez odczuwania bólu szyi?			
2. Czy możesz wykonywać codzienne czynności bez bólu szyi, ograniczającego twoją aktywność?			
3. Czy możesz wykonywać codzienne czynności bez pomocy innych?			
4. Czy poranne ubieranie się zajmuje Ci tyle samo czasu, co przedtem?			
5. Czy możesz pochylić się nad umywalką, aby umyć zęby, bez odczucia bólu?			
6. Czy spędzasz w domu więcej czasu, niż zwykle, z powodu bólu szyi?			
7. Czy ból szyi uniemożliwia ci podnoszenie przedmiotów ważących 2-5 kg?			
8. Czy z powodu bólu szyi musiałeś/aś ograniczyć czytanie?			
9. Czy odczuwasz ból głowy w momencie, kiedy odczuwasz ból szyi?			
10. Czy masz wrażenie, że z powodu bólu szyi zmniejszyła się twoja zdolność koncentracji?			
11. Czy z powodu bólu szyi nie możesz już spędzać wolnego czasu w typowy dla ciebie sposób?			
12. Czy z powodu bólu szyi zostajesz w łóżku dłużej niż zwykle?			
13. Czy masz wrażenie, że ból szyi wpłynął na twoje relacje z rodziną?			
14. Czy w ciągu ostatnich dwóch tygodni byłeś zmuszony zrezygnować z kontaktów towarzyskich z powodu bólu szyi?			
15. Czy masz wrażenie, że ból szyi wpłynie na twoją przyszłość?			

UWAGI:

____

____

____

BADAJĄCY: ____________________________
