# Supplementary material for: Cross-cultural adaptation of the Neck Disability Index and Copenhagen Neck Functional Disability Scale for patients with neck pain due to degenerative and discopathic disorders. Psychometric properties of the Polish versions
Source: BMC Musculoskelet Disord. 2011 Apr 29;12:84. doi: 10.1186/1471-2474-12-84 (PMC3108936; doi:10.1186/1471-2474-12-84)
Supplement: Additional file 2 — Neck Disability Index_Polish version. Polish language version of adapted Neck Disability Index [file 1471-2474-12-84-S2.PDF]

WSKAŹNIK NIEPEŁNOSPRAWNOŚCI SPOWODOWANEJ DOLEGLIWOŚCIAMI BÓLOWYMI CZĘŚCI SZYJNEJ KRĘGOSŁUPA

NECK DISABILITY INDEX-POLISH VERSION

Imię i nazwisko Pacjenta: ____ Data: ________

Proszę przeczytać polecenie:

Niniejszy kwestionariusz został przygotowany po to, by dostarczyć lekarzowi informacji o stopniu, w jakim dolegliwości bólowe części szyjnej kręgosłupa wpływają na zdolność wykonywania codziennych czynności. Odpowiedzi należy udzielić w każdej części, zaznaczając TYLKO JEDNO zdanie, które najbardziej odpowiada Państwa sytuacji. Zdajemy sobie sprawę, że do Państwa sytuacji może pasować więcej niż jedno zdanie, jednak PROSIMY O ZAZNACZANIE TYLKO JEDNEJ ODPOWIEDZI, KTÓRA NAJBLIŻEJ OPISUJE OBECNE DOLEGLIWOŚCI.

**CZĘŚĆ 1 – INTENSYWNOŚĆ BÓLU**	**CZĘŚĆ 6 – SKUPIENIE SIĘ**
□ Obecnie nie odczuwam żadnego bólu.	□ Mogę się w pełni skupić, kiedy chcę, bez problemu.
□ Obecnie ból jest bardzo łagodny.	□ Mogę się w pełni skupić, kiedy chcę, z małymi problemami.
□ Obecnie ból jest umiarkowany.	□ Skupienie się, kiedy chcę, sprawia mi pewne kłopoty.
□ Obecnie ból jest dosyć silny.	□ Skupienie się, kiedy chcę, sprawia mi duże kłopoty.
□ Obecnie ból jest bardzo silny.	□ Skupienie się, kiedy chcę, sprawia mi ogromne kłopoty.
□ Nie mogę sobie wyobrazić gorszego bólu, niż obecny.	□ W ogóle nie mogę się skupić
**CZĘŚĆ 2 – PIELĘGNACJA (MYCIE, UBIERANIE SIĘ, ITP.)**	**CZĘŚĆ 7 – PRACA**
□ Dbam o siebie normalnie i nie wywołuje to dodatkowego bólu.	□ Mogę pracować tyle, ile zechcę.
□ Dbam o siebie normalnie, ale wywołuje to dodatkowy ból.	□ Mogę wykonywać swoją normalną pracę, ale nie więcej.
□ Pielęgnacja sprawia mi ból, jestem powolny i delikatny.	□ Mogę wykonywać większość swojej normalnej pracy, ale nie więcej.
□ Potrzebuję trochę pomocy, ale większość rzeczy robię samodzielnie.	□ Nie mogę wykonywać swojej normalnej pracy.
□ Pod wszystkimi aspektami pielęgnacji osobistej potrzebuję codziennej pomocy	□ Prawie w ogóle nie mogę wykonywać żadnej pracy
□ Nie ubieram się, myję się z trudnością i zostaję w łóżku	□ W ogóle nie mogę wykonywać żadnej pracy
**CZĘŚĆ 3 – PODNOSZENIE PRZEDMIOTÓW**	**CZĘŚĆ 8 – PROWADZENIE SAMOCHODU**
□ Mogę podnosić ciężkie przedmioty i nie powoduje to dodatkowego bólu.	□ Nie odczuwam bólu szyi podczas prowadzenia samochodu.
□ Mogę podnosić ciężkie przedmioty, ale powoduje to dodatkowy ból.	□ Mogę prowadzić samochód tak długo jak tylko chcę, ale odczuwam delikatny ból szyi
□ Ból uniemożliwia mi podnoszenie dużych ciężarów z podłogi, ale mogę to zrobić, jeśli są one w wygodnym położeniu, np. na stole.	□ Mogę prowadzić samochód tak długo jak tylko chcę ale odczuwam umiarkowany ból karku.
□ Ból uniemożliwia mi podnoszenie dużych ciężarów, ale mogę to zrobić, jeśli średnie ciężary znajdują się w wygodnym położeniu.	□ Nie mogę prowadzić samochodu tak długo jakbym chciał, ponieważ odczuwam umiarkowany ból szyi.
□ Mogę podnosić tylko bardzo lekkie przedmioty.	□ Z powodu silnego bólu karku prawie w ogóle nie mogę prowadzić samochodu.
□ W ogóle nie mogę niczego podnosić ani nosić.	□ W ogóle nie mogę prowadzić samochodu.
**CZĘŚĆ 4 – CZYTANIE**	**CZĘŚĆ 9 – SPANIE**
□ Mogę czytać tyle ile zechcę i nie odczuwam bólu szyi	□ Nie mam problemów ze spaniem.
□ Mogę czytać tyle ile zechcę, ale odczuwam delikatny ból szyi	□ Mam niewielkie problemy ze spaniem (mniej niż 1 godz. bez snu).
□ Mogę czytać tyle ile zechcę, ale odczuwam umiarkowany ból szyi.	□ Mam małe problemy ze spaniem (1-2 godz. bez snu).
□ Nie mogę czytać tyle ile bym chciał/a, ponieważ odczuwam umiarkowany ból szyi.	□ Mam średnie problemy ze spaniem (2-3 godz. bez snu).
□ Prawie w ogóle nie mogę czytać, ponieważ odczuwam silny ból szyi.	□ Mam duże problemy ze spaniem (3-5 godz. bez snu).
□ W ogóle nie mogę czytać z powodu bólu szyi.	□ Mam ogromne problemy ze spaniem (5-7 godz. bez snu).
**CZĘŚĆ 5 – BÓL GŁOWY**	**CZĘŚĆ 10 – WYPOCZYNEK**
□ W ogóle nie odczuwam bólu głowy.	□ Mogę brać udział we wszystkich zajęciach rekreacyjnych, bez odczuwania bólu szyi.
□ Odczuwam delikatny ból głowy, który zdarza się sporadycznie	□ Mogę brać udział we wszystkich zajęciach rekreacyjnych, odczuwając lekki ból szyi.
□ Odczuwam umiarkowany ból głowy, który zdarza się sporadycznie.	□ Z powodu bólu szyi nie mogę brać udziału we wszystkich typowych dla mnie zajęciach rekreacyjnych
□ Odczuwam umiarkowany ból głowy, który zdarza się często.	□ Z powodu bólu szyi mogę brać udział tylko w kilku typowych dla mnie zajęciach rekreacyjnych
□ Odczuwam silny ból głowy, który zdarza się często.	□ Z powodu bólu szyi prawie w ogóle nie mogę brać udziału w typowych dla mnie zajęciach rekreacyjnych
□ Prawie cały czas odczuwam ból głowy.	□ W ogóle nie mogę brać udziału w jakichkolwiek zajęciach rekreacyjnych.
